# Expression of a Shiga-Like Toxin during Plastic Colonization by Two Multidrug-Resistant Bacteria, *Aeromonas hydrophila* RIT668 and *Citrobacter freundii* RIT669, Isolated from Endangered Turtles (*Clemmys guttata*)

**DOI:** 10.3390/microorganisms8081172

**Published:** 2020-08-01

**Authors:** Seema G. Thomas, Maryah A. Glover, Anutthaman Parthasarathy, Narayan H. Wong, Paul A. Shipman, André O. Hudson

**Affiliations:** Thomas H. Gosnell School of Life Sciences, Rochester Institute of Technology, Rochester, NY 14623, USA; sgtsbi@rit.edu (S.G.T.); mag3461@g.rit.edu (M.A.G.); axpsbi@rit.edu (A.P.); nhwsbi@rit.edu (N.H.W.); passbi@rit.edu (P.A.S.)

**Keywords:** *Citrobacter*, *Aeromonas*, biofilm, turtle, Shiga-like toxin, antibiotic resistance, plastic, whole-genome sequencing

## Abstract

*Aeromonas hydrophila* RIT668 and *Citrobacter freundii* RIT669 were isolated from endangered spotted turtles (*Clemmys guttata*). Whole-genome sequencing, annotation and phylogenetic analyses of the genomes revealed that the closest relative of RIT668 is *A. hydrophila* ATCC 7966 and *Citrobacter portucalensis* A60 for RIT669. Resistome analysis showed that *A. hydrophila* and *C. freundii* harbor six and 19 different antibiotic resistance genes, respectively. Both bacteria colonize polyethylene and polypropylene, which are common plastics, found in the environment and are used to fabricate medical devices. The expression of six biofilm-related genes—biofilm peroxide resistance protein (*bsmA*), biofilm formation regulatory protein subunit R (*bssR*), biofilm formation regulatory protein subunit S (*bssS*), biofilm formation regulator (*hmsP*), toxin-antitoxin biofilm protein (tabA) and transcriptional activator of curli operon (*csgD*)—and two virulence factors—Vi antigen-related gene (*viaB*) and Shiga-like toxin (*slt*-*II*)—was investigated by RT-PCR. *A. hydrophila* displayed a > 2-fold increase in *slt-II* expression in cells adhering to both polymers, *C. freundii* adhering on polyethylene displayed a > 2-fold, and on polypropylene a > 6-fold upregulation of *slt-II*. Thus, the two new isolates are potential pathogens owing to their drug resistance, surface colonization and upregulation of a *slt-II*-type diarrheal toxin on polymer surfaces.

## 1. Introduction

Antibiotic resistance is an increasing crisis as both the range of resistance in clinical settings expands and the pipeline for development of new antibiotics contracts [1]. This problem is compounded by the global genomic scope of the antibiotic resistome, so that antibiotic resistance spans a continuum from genes in clinical pathogens to those of benign environmental microbes along with their proto-resistance gene progenitors [2,3]. Further, increased resistance to antimicrobial agents occurs in biofilms [4]. Biofilm-associated cells differ from their suspended counterparts by generation of an extracellular polymeric substance (EPS) matrix, reduced growth rates, and the up/downregulation of specific genes [5]. Plasmid conjugation occurs at a greater rate between cells in biofilms than between planktonic cells [6,7,8]. Bacterial biofilms constitute a serious problem for public health due to their potential to colonize in-dwelling medical devices (IMDs) [9,10], such as abdominal [11] and coronary stents [12], which contain polymeric materials such as polyethylene (PE) and polypropylene (PP). Once infected, the IMDs are often removed and replaced, causing a significant increase in the health care cost and chance of reinfection [13]. Similarly, water supplies contaminated with biofilms are a significant risk for public health [14].

Several studies reported the existence of this plastic-specific microbial community in aquatic systems [15,16,17,18,19,20,21]. For instance, the microbial community composition of the biofilm developing on submerged wood, glass and cellulose differed from that on PE and PP [16]. Characklis et al. [22], noted that the extent of microbial colonization appears to increase with surface roughness, and other reports show that microorganisms attach more rapidly to hydrophobic, non-polar surfaces such as Teflon and other plastics than to hydrophilic materials such as glass or metals [23,24,25]. The type of polymer influences the microbial community composition of the associated biofilm. For example, keratin-based biopolymers are being extensively explored for biomedical applications [26,27,28]. However, they may also be more susceptible to bacterial colonization, since Proteobacteria have been shown to colonize keratin-rich surfaces such as the shells of freshwater turtles [29]. Success in the war against biofilms requires a deeper understanding of the interactions between biofilm cells, the surface, antibiotics, and the host [30,31].

*Aeromonas* species are Gram-negative bacteria found in aquatic environments, soil, sewage and sediments. They are regarded as opportunistic pathogens of humans and other animals, especially fish. They can cause gastroenteritis, bacteraemia, peritonitis and infections of the respiratory system, hepato-biliary system, urinary tract, eyes and wounds [32,33]. Necrotizing fasciitis has also been reported with *A. hydrophila* as the causative agent [34,35]. Outbreaks of diarrhea have also been reported [36]. Most aeromonads have type IV pili, which facilitate horizontal gene transfer via conjugation of mobile genetic elements between different bacterial species [37]. Shiga-type toxins were reported from clinical and environmental *Aeromonas* isolates, with many having highly sequence similarity to homologs in virulent *Escherichia coli* strains [38,39]. Other studies show that up to 73% of the environmental strains may be naturally competent for taking up extracellular DNA [40]. Acquired resistance increases the multidrug-resistant character of both environmental and clinical strains of *Aeromonas* [41]. Extended-spectrum β-lactamase genes were transferred via plasmids from enterobacteria (possibly from the human gut) to *Aeromonas* [42]. Transposons, integrons and plasmids may also confer resistance to β-lactams, quinolones, macrolides, tetracyclines, sulfonamides, and chloramphenicol [32,43].

Recent studies have indicated that although *Aeromonas* spp. cause a range of pathologies, they are emerging as an enteric pathogen of public health concern [44]. Results from diarrheal outbreak studies further show that the infective dose of *Aeromonas* is very low [45]. *A. hydrophila* has also been found in food samples and has the ability to form mixed biofilms with other pathogens [46]. Aeromonads colonize the surfaces and insides of plants and animals [47], as well as abiotic surfaces like sediment, steel, glass, and polyvinyl chloride [48,49,50,51]. Notably, *Aeromonas* spp. attach to surfaces and eventually form biofilms even when freely able to grow in water, since this may enable long-term persistence in aquatic environments [52]. In natural systems, these persisting adherent cells were genetically distinct from the cells which were actually free-floating in the water column [53]. Biofilms, however, provide an ideal niche for the exchange of plasmids [6,7,8]. Studies have already noted the expression of similar Shiga-toxins in both environmental and clinical isolates of *Aeromonas* [39]. The relative abundance of *Aeromonas* spp. increased in riverine microplastics, suggesting that they could use plastics as vectors [20]. Recent studies have demonstrated that cells adhering to surfaces but not inside biofilms have resistance profiles similar to biofilm cells [54,55].

*Citrobacter freundii* is a member of the genus *Citrobacter*, belonging to the Gram-negative family Enterobacteriaceae [56,57,58]. It is found in soil, water, food and as a commensal in the gastrointestinal tract of humans and other animals [56,57,59]. However, it is increasingly a nosocomial and environmental pathogen, causing pneumonia, diarrhea, urinary tract and bloodstream infections [59,60,61,62,63,64,65,66]. Food poisoning cases caused by *C. freundii* isolates have been reported [59,62]. Cases of neonatal infections in preterm infants are also known [67]. Antibiotic-resistant *C. freundii* strains have been increasing around the world, and extended β-lactamase and plasmid-mediated quinolone resistance have been documented [66,68,69,70,71,72]. *C. freundii* strains have been isolated from mixed biofilms from water supply systems, growing along with strains such as *Agrobacterium tumefaciens*, *A. hydrophila*, *Enterobacter soli* and *Stenotrophomonas maltophilia* [73]. The presence of *C. freundii* worsens existing *Pseudomonas aeruginosa* infections in murine models and also likely in patients with co-infection [74]. Further, biofilm infections containing enteroaggregative *E. coli* and aggregative *C. freundii* cause diarrhea; in this case, the interaction is mediated by putative F pili [75]. Foodborne outbreaks, hospital and pediatric outbreaks, some involving hemolysis in addition to diarrhea, have also been reported [76,77,78]. Genomic analysis of a cytotoxic and aggregative strain of *C. freundii* revealed that it had acquired potential virulence factors including 7 genomic islands, 2 fimbriae islands and a type VI secretion system island [59]. Major virulence factors from human isolates were described as Shiga-like toxins and heat-stable toxins [77,79]. Later studies showed that an 18-amino acid peptide of a clinical *C. freundii* isolate was identical to that of an *E. coli* Shiga-toxin [79,80]. Other studies also reported Shiga-like toxins with 99.5–100% sequence similarity to *E. coli* toxins [81]. A cholera toxin B subunit homolog was also reported from a clinical strain [82].

Reptile-associated bacteria may be capable of infecting warm-blooded mammals, since there is a previous case of reptile and clinical strains, including *Aeromonas* spp., being identical [83]. Recent zoonotic outbreaks such as the SARS-CoV-2 pandemic [84] underscore the need to assess and identify potential future pathways for animal–human disease transmission. Both *A. hydrophila* and *C. freundii* are documented pathogens found among both freshwater and marine chelonians, and include antibiotic-resistant forms [85,86,87]. The study of antibiotic-resistant bacteria in marine turtles may also be used as a bio-indicator of exposure to effluents and other sources of environmental contamination [88,89]. Thus, turtles are a “sentinel species” for ecosystem health [90,91]. The often illegal and unregulated trade of reptiles sold at “wet markets” worldwide poses serious human health threats [92,93]. Physiologically stressed freshwater and marine reptiles are most often maintained in unsanitary conditions in plastic and wooden enclosures, providing ideal conditions for disease-causing bacteria and the formation of biofilms. Recent studies raised the possibility that immune-compromised turtles may be harboring and expressing significant pathogenic potential in the gut, and contribute to spreading them in the marine environment [90,94,95]. It is already known that marine turtles ingest microplastics [96] and that microplastics promote gene exchange and the occurrence of integrase genes such as *int1* [97,98]. Connecting the dots, *A. hydrophila* and *C. freundii* could inhabit freshwater and marine reptiles and colonize the plastics they ingest. Then, highly drug-resistant biofilm forms of these bacteria could grow on the plastic surfaces within the gut and may be released by feces and contaminate water/food and eventually cause human disease. The possible colonization of plastic surfaces of water piping and IMD by turtle-derived *A. hydrophila* and *C. freundii* may carry additional risks of infection.

The aim of this study was to predict the potential for antibiotic resistance of our isolates based on whole-genome sequencing. Apart from antibiotic resistance genes, biofilm formation and the presence of toxins increase the damage any potential pathogen may cause. Therefore, we aimed to study the expression of six selected biofilm-related genes (*bsmA*, *bssR*, *bssS*, *hmsP*, *tabA* and *csgD*), and the virulence-associated *viaB* and *slt-II* during planktonic and adherent growth with PE and PP surfaces, after a period of 6 weeks. Genome analysis of our strains shows that both of them are potentially multidrug resistant. Further, we show that the expression of *slt-II* is enhanced in the adherent growth phase for both organisms on either PE or PP. We suggest that the predicted extensive antibiotic resistance and the ability to express a Shiga-like toxin warrants the classification of both strains as potential zoonotic opportunistic pathogens. Finally, the adherent phenotypes generated in this study were distinct compared to classical biofilms induced by the presence of blood/bile components, when analyzed by scanning electron microscopy.

## 2. Materials and Methods

### 2.1. Bacterial Isolation

Microbial samples were isolated from 12 adult rescued infected spotted turtles (*Clemmys guttata*) seized by the United States Fish and Wildlife Service from an illegal reptile trade operation (chain of custody ID number-ST#032797). The spotted turtle is a small, semi-aquatic, North American species commonly targeted and illegally harvested for sale in the pet trade and overseas for other uses [99,100]. The eyes, nostrils and limbs of turtles were swabbed on to agar plates and the samples were initially subjected to biochemical assays. For subsequent analysis, the two strains identified as *Aeromonas hydrophila* RIT 668 and *Citrobacter freundii* RIT 669 (based on 16S rDNA sequences) were routinely cultured on blood agar plates (5% sheep blood) and MacConkey plates respectively (BD BBL™, prepared media, 100 mm × 15 mm, San Diego, CA, USA). Hemolysis on the blood plates was examined by observing the presence of complete lysis around the colonies and a clearing on the medium. All twelve turtles (*Clemmys guttata*) were infected, lethargic and had reduced/stopped food intake for around a week. The eyes, nostrils and feet of the spotted turtles were surrounded by a slimy substance, and the eyes were inflamed. All the turtles were infected by *A. hydrophila* and *C. freundii*.

### 2.2. Characterization and Identification: Biochemical Assay and 16S rDNA Amplification

Primary identification was achieved via Gram staining. Oxidase and catalase tests were performed, followed by five groups of biochemical assays for microbial identification: Group 1—glucose, gas, and lysine; Group 2—ornithine, H_2_S, and indole; Group 3—adonitol, lactose, and arabinose; Group 4—sorbitol, Voges Proskauer, and dulcitol; and Group 5—phenylalanine (PA), urea, and citrate. Of the four different microbes identified, two strains were further analyzed and identified via 16S rDNA PCR amplification. For the 16S rDNA amplification, the microbial DNA was isolated using the ‘UltraClean Microbial DNA Isolation Kit’ (*MO BIO* Laboratories Inc., San Diego, CA, USA). Colony PCR was performed to obtain ~500 base pair amplicons of the 16S V3/V4 regions of the rRNA gene using the following conditions: 1 cycle at 95 °C for 2 min, followed by 30 cycles at 95 °C for 30 s, 52 °C for 30 s, and 72 °C for 3 min. The forward and reverse primers used for this was 5′-CCTACGGGNGGCWCGAG-3′ and 5′-GACTACHVGGGTATCTAATCC-3′. The amplicons were separated by gel electrophoresis, followed by commercial Sanger nucleotide sequencing (GeneWiz LLC, South Plainfield, NJ, USA) using the V3/V4 forward primer.

### 2.3. Genomic DNA Isolation

Genomic DNA was extracted from mid-log phase of *Aeromonas hydrophila* RIT668 and *Citrobacter fruendii* RIT669 using the GenElute Bacterial Genomic DNA kit, as per the manufacturer’s protocol (Sigma-Aldrich, St. Louis, MO, USA), and quantified using a Nanodrop One spectrophotometer.

### 2.4. Agarose Gel Electrophoreses

The DNA fragments from PCR and RT-PCR experiments were run on 1% agarose gels submerged in 1X TAE buffer, and stained with ethidium bromide, followed by visualization with a UV light source. A 1 kb ladder was used as the DNA standard (New England Biolabs, Ipswich, MA, USA).

### 2.5. Whole-Genome Sequencing, Assembly and Annotation

Using the Nextera XT library prep kit (Illumina) and Nextera XT index kit (Illumina), 1 ng of genomic DNA was processed to generate a sequencing-ready library, as per the manufacturer’s protocol. After library prep, 5 µL tagmented and indexed library DNA was quantified via the PicoGreen broad range assay on a Biotek Synergy H1 plate reader. To determine the fragment size distribution of DNA in the tagmented and indexed library, 1 µL of DNA was run using the DNA 1000 chip kit on an Agilent 2100 bioanalyzer. Based on the DNA concentration and size distribution, the library was manually normalized to a concentration of 4 nM in molecular-grade water. Of the resulting 4 nM library, 5 µL was then pooled with other tagmented, indexed, and normalized libraries. The pooled libraries were denatured and diluted to a loading concentration of 12 pM following the manufacturer’s protocol for manually normalized libraries. The pooled, denatured and diluted 12 pM library was sequenced using the MiSeq Reagent Kit V3 on the Illumina MiSeq for 2 × 151 cycles at the Rochester Institute of Technology Genomics Facility. Adapter trimming was performed automatically on the Illumina MiSeq during FASTQ generation. Trimmed reads were uploaded to the Galaxy web platform, and assembled de novo at the public server at usegalaxy.org [101], using Unicycler version 0.4.6.0 [102], with a minimum contig length of 200 bp.

### 2.6. Phylogenetic Analysis

The assembled FASTA contig files for *A. hydrophila* RIT668 and *C. freundii* RIT669 were uploaded to the Type Strain Genome Server (TYGS), found at https://tygs.dsmz.de. TYGS is a freely available tool for creating taxonomic assignments based on whole-genome sequence data [103]. The ten closest strains in the TYGS database to the query assembly were determined using Mash, a whole-genome clustering method [104]. Additionally, another ten closest relatives to the query assembly were selected by BLAST comparison of the 16S rDNA sequences extracted from the query using RNAmmer [105] against all 10,342 type strains in the TYGS database [106], selecting the top 50 hits, and calculating the Genome BLAST Distance Phylogeny distance (GBDP) with the query genome to determine the closest 10 type strains [107].

### 2.7. Resistance Gene Identifier (RGI)

The antibiotic resistance genes in both the model organisms were identified using the Resistance Gene Identifier (RGI) tool for understanding the mechanisms of antimicrobial resistance. The AMR gene family was identified along with the drug class, percentage identify of matching region and length of reference sequence. The genomes of *A. hydrophila* and *C. freundii* were analyzed using the bioinformatics platform RGI CARD. FASTA files were uploaded to RGI CARD software server RGI 5.1.0, CARD 3.0.4 [108] using the following parameters: Perfect, Strict and Loose hits, and complete genes only. The Perfect algorithm is most useful for clinical surveillance as it detects perfect matches to the curated reference sequences and mutations in the CARD. The Strict algorithm is aimed at unearthing previously unknown variants of known antibiotic resistance genes, including secondary screen for key mutations.

### 2.8. Predictions of Secondary Metabolite Production

The assembled genome sequence of *A. hydrophila* RIT668 and *C. freundii* RIT669 were analyzed using the Antibiotics and Secondary Metabolite Analysis Shell (antiSMASH5.0) webserver [109]. This tool identifies the biosynthetic loci covering the whole range of known secondary metabolite compound classes. It aligns the identified regions at the gene cluster levels to the closest match from a database, which contains all other known gene clusters and integrates the previously available secondary metabolite-related genes.

### 2.9. Colonization of Planktonic and Biofilm Forms on Plastics

#### 2.9.1. Classical Biofilm Forms

The colonization of polyethylene (PE) and polypropylene (PP) plastics was studied using the two model organisms. High-density 1.6 mm PE and PP sheets were laser cut to 1 inch squares and sterilized with alcohol followed by UV radiation for 3 h. Sterilized squares were placed aseptically in blood agar plates and MacConkey plates, and inoculated with 5 mL each of overnight culture of *A. hydrophila* and *C. freundii*, respectively, for biofilm formation. The plates were incubated at 37 °C for 6 weeks with a steady supply of 3 mL of liquid bacterial cultures and analyzed for plastic biofilm colonization via scanning electron microscopy (SEM). This set up in solid agar media is referred to as ‘biofilms’ throughout the study and was used only for SEM comparisons.

#### 2.9.2. Planktonic and Adherent Forms

A single plastic PE or PP square was placed in each well in a six-well dish with 5 mL of *C. freundii* or *A. hydrophila* culture with an OD_600_ of 0.5. Planktonic cells were aspirated out every other day in order to replace the well with fresh TSB depending on the dish. Dishes were incubated at 37 °C. Controls were initially placed alongside cultures in a six-well dish. To allow for maximum possible plastic colonization, except for aspiration of old TSB media and addition of new media, the six-well dish was left undisturbed for the duration of 6 weeks. This set up in liquid media is referred to as ‘planktonic’ throughout the study and the adherent cells as ‘adherent’.

#### 2.9.3. RNA Isolation

RNA was isolated from the *A. hydrophila* and *C. freundii* samples using the Omega E.Z.N.A. bacterial RNA isolation kit as per the manufacturers’ protocol (Omega Bio-tek Inc., Norcross, GA, USA). The RNA integrity was analyzed NanoDrop followed by running the samples in a 1.5% agarose gel alongside a 1 Kb ladder (Invitrogen, Carlsbad, CA, USA). An array of nine different annealing temperature were tested on eight different primers in order to elucidate and optimize the temperatures for each of the samples.

### 2.10. Detection of Biofilm-Related Genes and Virulence Factors by RT-PCR

RT-PCR was performed based on the primer sequences and melting temperatures listed in Table 4. The genes encoding the following proteins were analyzed (a) Biofilm-related BsmA (biofilm peroxide resistance protein), BssR (biofilm formation regulatory protein), BssS (biofilm formation regulatory protein), HmsP (biofilm formation regulator), TabA (toxin-antitoxin biofilm protein) and CsgD (transcriptional activator curli operon). (b) Virulence-related: ViaB (primers VIAB-1 and VIAB-2, expected product size 516 bp; [110]), and Shiga-like toxin II (SLT-II) using the published primers GK1 and GK4, designed to amplify *slt*-II genes with an expected product size of 1260 bp [81,111].

A reverse transcriptase system (2 step) with cDNA synthesis (Promega Corp., Madison, WI, USA) was used for RT-PCR followed by the thermal cycle program. Since nine different annealing temperatures were tested in order to optimize the temperatures for each of the samples and each primer, the thermal cycle program varied for the primers and samples used in this study. A sample thermal cycle program involved initial denaturation at 95 °C for 3 min ×denaturation, annealing and extension at varying temperatures for 1 min (35 cycles) and 72 °C for 1 min respectively, followed by a final extension at 72 °C for 5 min and a final hold at 4 °C.

### 2.11. Estimation of Gene Expression

The change in gene expression was analyzed by running electrophoresis gels with sample concentrations normalized. Then, each set of bands corresponding to amplified fragments was analyzed for intensity and peak area with background subtraction, using the scientific image analysis open platform ImageJ [112]. The values were then plotted in Microsoft Excel as bar graphs showing fold change normalized to the 16S gene values, in the form of bar graphs. The 16S gene was used as the housekeeping standard during the RT-PCR and its values were used in this analysis.

### 2.12. Scanning Electron Microscopy Analysis

The biofilm-covered PE and PP squares were gently rinsed with phosphate-buffered saline (PBS) buffer at pH 7.4 and 2% glutaraldehyde to fix the cells and then incubated at 25 °C for 90 min. The samples were dehydrated with ethanol, incubated, and rinsed successively (50% for ten minutes, 70% for ten minutes, 80% for ten minutes, 95% twice for ten minutes, and 100% three times for fifteen minutes) inside a Petri dish. All the liquid was removed and the Petri dish containing the samples was covered in parafilm and stored at 25 °C until further analysis. Samples were covered with gold-palladium for two minutes with an SPI sputter coater to mitigate charging in the electron beam. The SEM was performed at 5 kV using a Mira3Tescan field emission SEM at the Rochester Institute of Technology (RIT) Nano-Imaging Lab.

## 3. Results

### 3.1. Biochemical Characterization and Taxonomy

**Figure 1:**
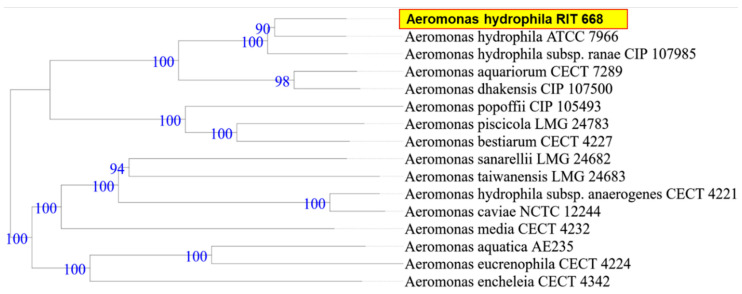
WGS-based phylogenetic tree for *A. hydrophila* RIT668, using the TYGS platform. TYGS infers trees with FastME 2.1.4 [103] based on Genome BLAST Distance Phylogeny (GBDP) distances calculated from the genome sequences. Branch lengths are scaled in terms of GBDP distance formula d5 [107]. Numbers above branches are GBDP pseudo-bootstrap support values from 100 replications.

**Figure 2:**
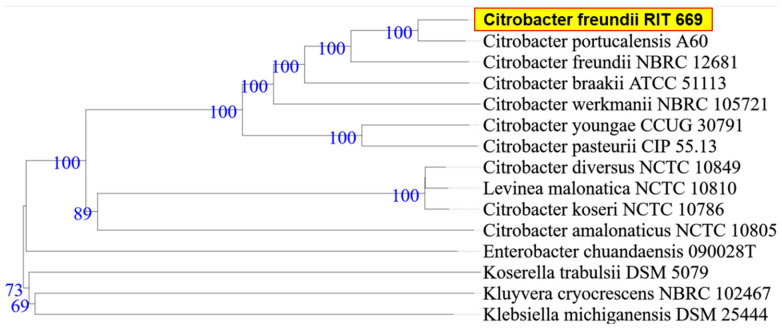
WGS-based phylogenetic tree using TYGS for *C. freundii* RIT669. TYGS infers trees with FastME 2.1.4 [103] based on Genome BLAST Distance Phylogeny (GBDP) distances calculated from the genome sequences, whereas branch lengths are scaled in terms of GBDP distance formula d5 [107]. Numbers above branches are GBDP pseudo-bootstrap support values from 100 replications.

When the research was begun, there was 50% mortality, in spite of following appropriate animal care. By the end of the study period, however, all twelve turtles died (mortality rate 100%). Microbial isolates from rescued spotted turtles (*Clemmys guttata*) were identified as *A. hydrophila* and *C. freundii*, and were Gram-negative, beta hemolytic, lactose fermenting, and potential opportunistic pathogens. The two strains were initially identified through the 16S rDNA sequence coupled with NCBI-BLAST searches. Owing to their possible pathogenicity, the genomes were sequenced and annotated; a summary of the genome characteristics is shown in Table 1. Taxonomy based on whole-genome comparisons of the ten nearest relatives using the TYGS tool [103] confirmed the identification of RIT668 as *A. hydrophila* and RIT669 as *Citrobacter.* The nearest relative of *A. hydrophila* RIT668 is *A. hydrophila* ATCC 7966 (Figure 1). *C. freundii* RIT669 is the closest relative of *Citrobacter portucalensis* A60 (Figure 2).

### 3.2. Resistome Analysis and Secondary Metabolite Analysis

According to the Resistance Gene Identifier (RGI) analysis of its genome, *A. hydrophila* RIT668 is potentially resistant to the carbapenem, penem, cephalosporin, fluroquinolone, tetracycline and elfamycin antibiotic classes (Table 2). It has homologs with 43–99.7% sequence identities to known examples of multidrug, unclassified, MLS (macrolide, lincosamide, streptogramin), aminoglycoside, β-lactam, bacitracin and glycopeptide resistance genes, with putative efflux pumps present. *C. freundii* RIT669 potentially resists up to 19 different classes of antibiotics, including carbapenem, penem, cephalosporin, fluroquinolone, tetracycline, peptides and elfamycin; mutilple efflux pumps are present (Table 3). Notably, it may also be resistant to classes such as fosfomycin, rhodamine, triclosan and benzalkonium chloride (the last two are widely used in consumer and sanitizer products). The closest relative *C. portucalensis* A60 is also aquatic and harbors resistance to β-lactams and quinolones [113].

### 3.3. Polymer Adhesion and Gene Expression

**Figure 3:**
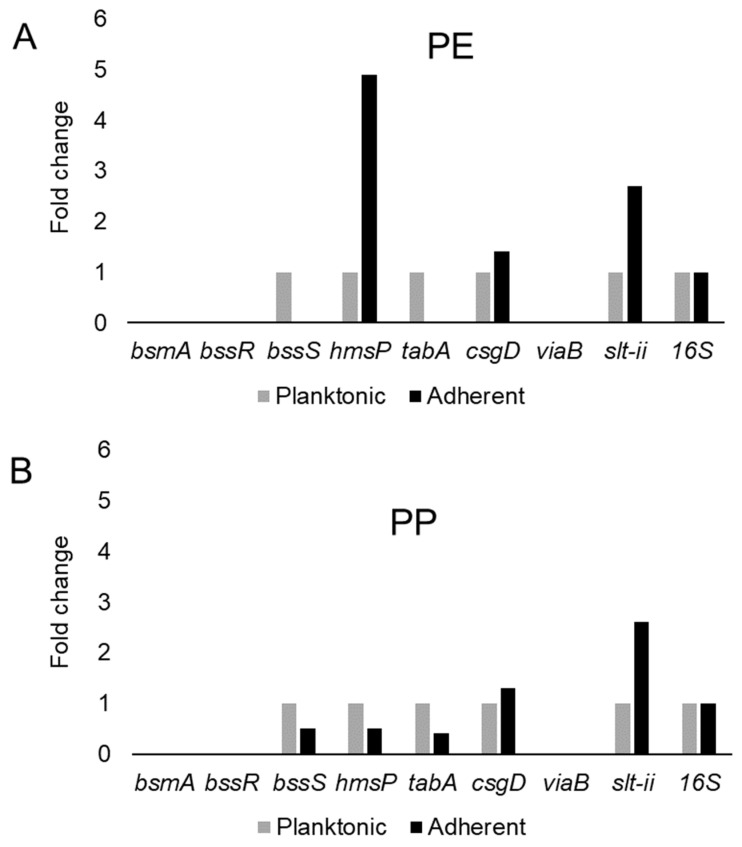
Variation in gene expression during planktonic and adherent growth of *A. hydrophila* using tryptic soy broth on polyethylene (PE) surface as shown in (**A**) and polypropylene (PP) surface as shown in (**B**), respectively. The 16S rRNA gene expression was used as an internal control.

**Figure 4:**
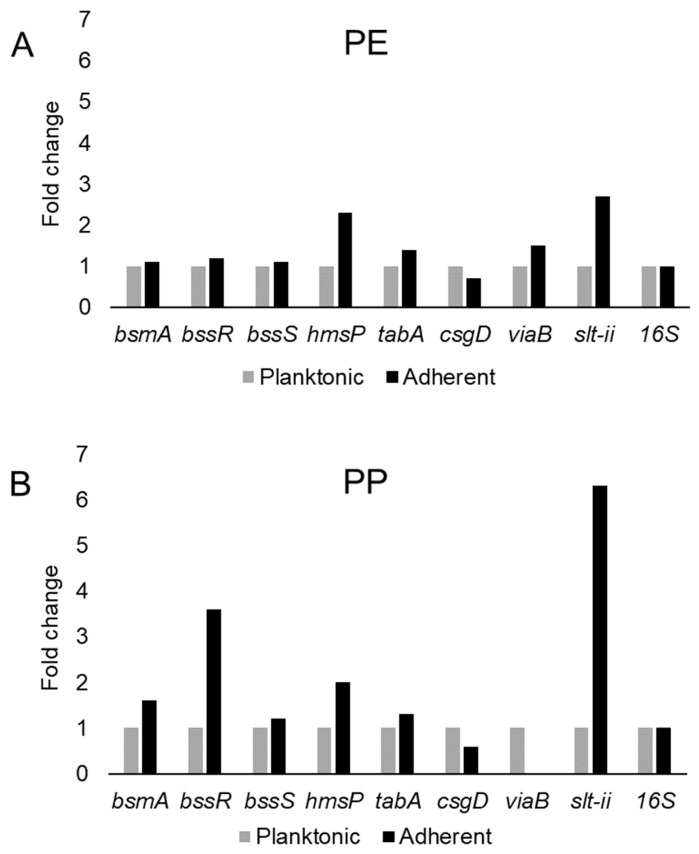
Variation in gene expression during planktonic and adherent growth of *C. freundii* in tryptic soy broth on polyethylene (PE) surface as shown in (**A**) and polypropylene (PP) surface as shown in (**B**), respectively. The 16S rRNA gene expression was used as an internal control.

This study examined the expression of six biofilm-related genes as well as *slt-II* and *viaB*, based on the primers listed in Table 4, which were derived from earlier studies [81,85,114,115]. The genes *bsmA*, *bssR*, *bssS* and *csgD* were reported to contribute to planktonic and biofilm growth in *Citrobacter werkmanii* BF6 [114]. The genes *tabA* and *hmsP* were identified as biofilm related in a subsequent study on the same organism [115]. The gene *bsmA* (biofilm stress and motility) or biofilm peroxide resistance protein, known as *yjfO* in *E. coli*, is upregulated during biofilm growth [116] Mutant analysis revealed roles in microcolony formation, flagellar motility as well as resistance to acid and peroxide stresses [116]. It has been suggested that *bsmA* is involved in controlling cell aggregation at specific points in biofilm development [116,117]. The genes *bssR* and *bssS* (regulator of biofilm through signal secretion), also called *yliH* and *yceP* in *E. coli* K-12, were shown to repress motility by mediating cell signaling [118]. Both *bssR* and *bssS* were postulated to be global regulators of several genes involved in catabolite repression, stress response, quorum sensing and the putative stationary-phase signal [118]. *A. hydrophila* samples did not amplify PCR products corresponding to *bsmA* and *bssR.* However, planktonic cells showed the amplification of *bssS*, while cells adhering on PE did not express *bssS* and adherent cells on PP appeared to repress *bssS* expression (Figure 3). *C. freundii* expressed *bsmA*, *bssR* and *bssS*; most were slightly upregulated in adherent cells on both polymers, but *bssR* was upregulated over 3.5-fold in the PP-adherent cells (Figure 4).

The gene *hmsP* (biofilm formation regulator) is one of two genes controlling the amount of biofilm produced in the pathogen *Yersinia pestis* via possible phosphodiesterase activity to modulate the levels of cyclic nucleotides [119]. It was shown that the EAL domain is essential for the biofilm inhibition activity of *hmsP* [120]. For *A. hydrophila*, *hmsP* was 4-fold upregulated in adherent cells on PE, but downregulated upon adherence to PP (Figure 3). *C. freundii* upregulated *hmsP* about 2-fold upon adherence to either PE or PP (Figure 4). The toxin-antitoxin gene *tabA*, known as *yjgK* in *E. coli*, influences biofilm formation in a time-dependent manner [121]. During the early phase *tabA* expression suppresses biofilm formation, while in later phases, it increases biofilm formation; it also suppresses biofilm dispersal [121]. PE-adherent *A. hydrophila* did not amplify *tabA*, but PP-adherent samples showed a slight downregulation (Figure 3). *C. freundii* showed a slight upregulation of *tabA* upon attachment to either polymer (Figure 4).

The curli operon-related gene *csgD* is a master regulator of biofilm formation and activates the synthesis of curli fimbriae and EPS in *E. coli*; *csgD* suppresses cell motility, triggering biofilm formation [122]. A homolog of *csgD* is expressed in the irreversible step of biofilm formation in *Actinobacillus pleuropneumoniae* [123]. Bacterial invasion of host cells may be controlled by c-di-GMP via *csgD* [124]. Cellulose biosynthesis is promoted by *csgD*, which is the transcriptional activator for curli production [125]. For both polymers, adherent *A. hydrophila* showed slightly upregulated expression of *csgD* (Figure 3), whereas adherent *C. freundii* downregulated *csgD* (Figure 4). The Vi antigen is commonly found in strains of *Salmonella enterica* serovars Typhi and Paratyphi, where it contributes to bacterial virulence and pathogenesis [126,127,128]. However, *viaB*-related genes for parts of the Vi antigen, which may contribute to invasiveness, have also been reported from *Salmonella enterica* serotype Dublin, *E. coli* and *Citrobacter* strains [129,130]. *A. hydrophila* samples did not amplify *viaB* (Figure 3), but slight upregulation is seen in PE-adhering *C. freundii* cells, whereas the PP-adhering cells did not amplify this gene (Figure 4). *A. hydophila* showed more than 2-fold upregulation of *slt-II* upon attachment to PE or PP (Figure 3). PE-adhering *C. freundii* upregulated *slt-II* expression more than 2-fold, while PP-adhering cells upregulated the expression of the same gene more than 6-fold (Figure 4). Biofilm-related genes were not upregulated and the lack of correlation between the expression of biofilm-related genes and the toxin genes remains unexplained. In *Escherichia coli* O104:H4 expressing the *stx2* gene, the correlation of the expression of other genes such as *pgaA* and *aggR* with *stx2* expression is strain dependent [131].

### 3.4. Secondary Metabolite Production via antiSMASH

Genome mining for secondary metabolite-producing gene clusters using the antiSMASH tool (108) showed that *A. hydrophila* has the potential to produce non-ribosomal peptides (NRP), arylpolyene, homeserine lactone and bacteriocin type of molecules (Table 5). *C. freundii*, on the other hand, possesses two biosynthetic gene clusters predicted to produce arylpolyene and NRP-type molecules (Table 5). The *A. hydrophila* gene clusters have high similarity to those of other aeromonads. Interestingly, homeserine lactone signals are known to coordinate quorum sensing and biofilm formation in many Gram-negative bacteria [132,133,134].

### 3.5. Electron Microscopy Analysis

**Figure 5:**
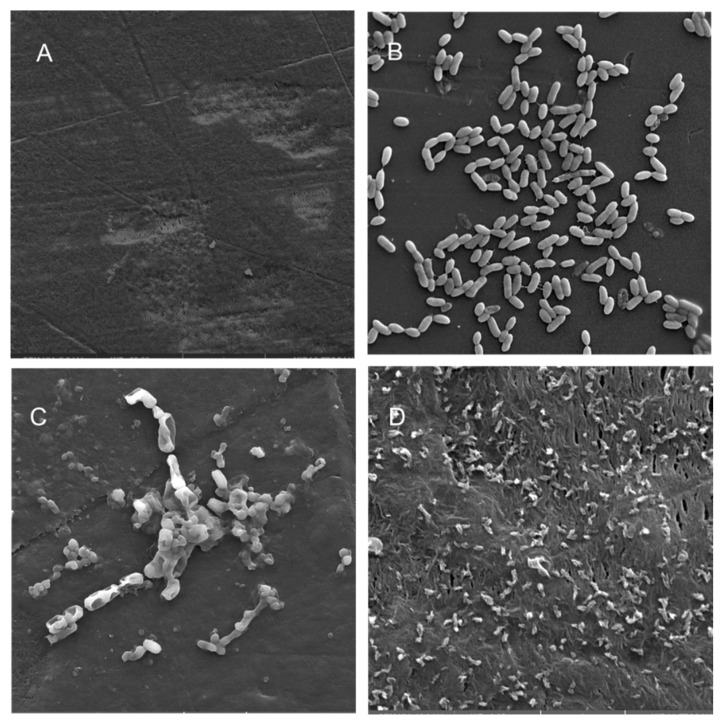
Scanning electron micrographs (**A**) of un-colonized PE (×4980); (**B**–**D**) of *A. hydrophila* in the planktonic phase (×6470), adherent phase on PE (×15,900) and biofilm phase induced on PE pressed on blood agar (×19,700), respectively.

**Figure 6:**
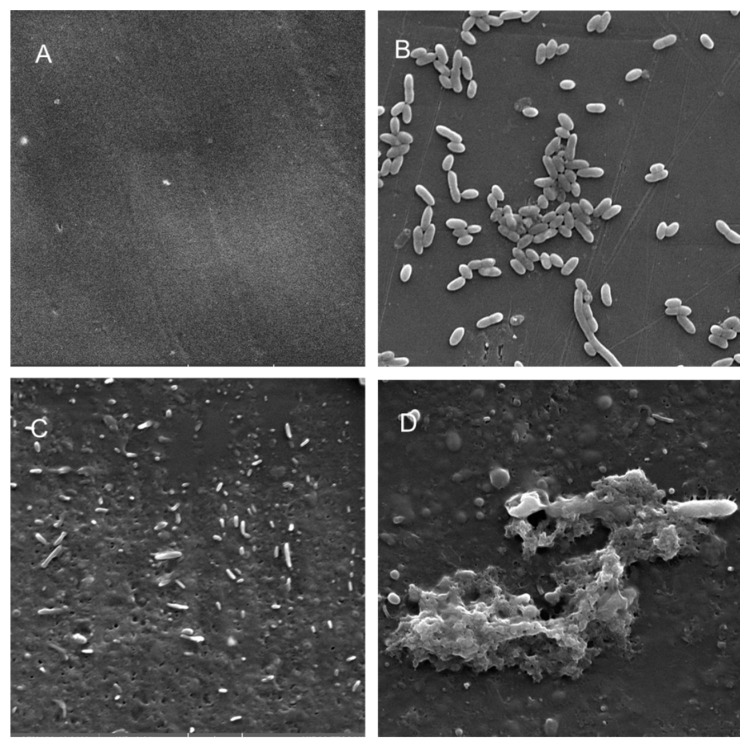
Scanning electron micrographs (**A**) of un-colonized PP (×10,000); (**B**–**D**) of *A. hydrophila* in the planktonic phase (**B**; ×6540), adherent phase on PP (**C**; ×7530) and biofilm phase induced on PP pressed on blood agar (**D**; ×2540).

**Figure 7:**
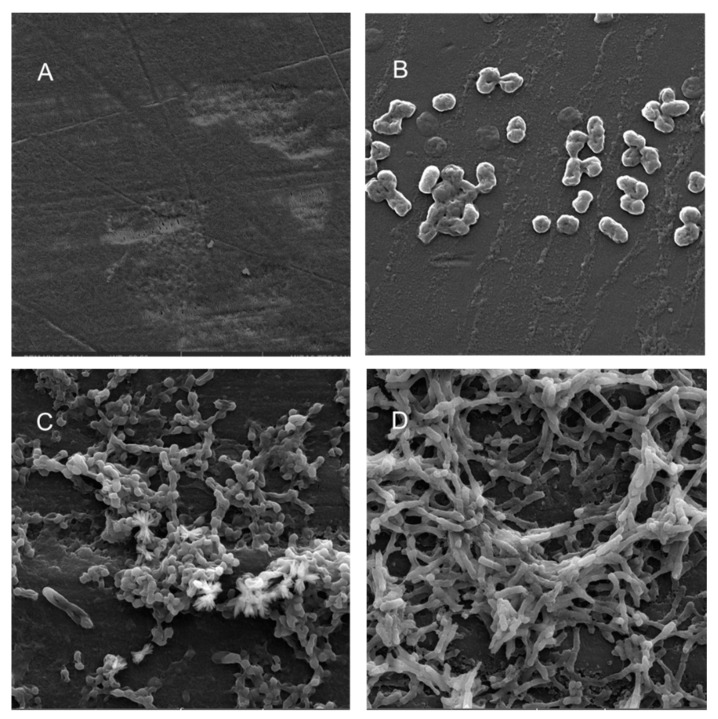
Scanning electron micrographs (**A**) of un-colonized PE (×4980); (**B**–**D**) of *C. freundii* in the planktonic phase (×16,800), adherent phase on PE (×10,000) and biofilm phase induced on PE pressed on MacConkey agar (×8300), respectively.

**Figure 8:**
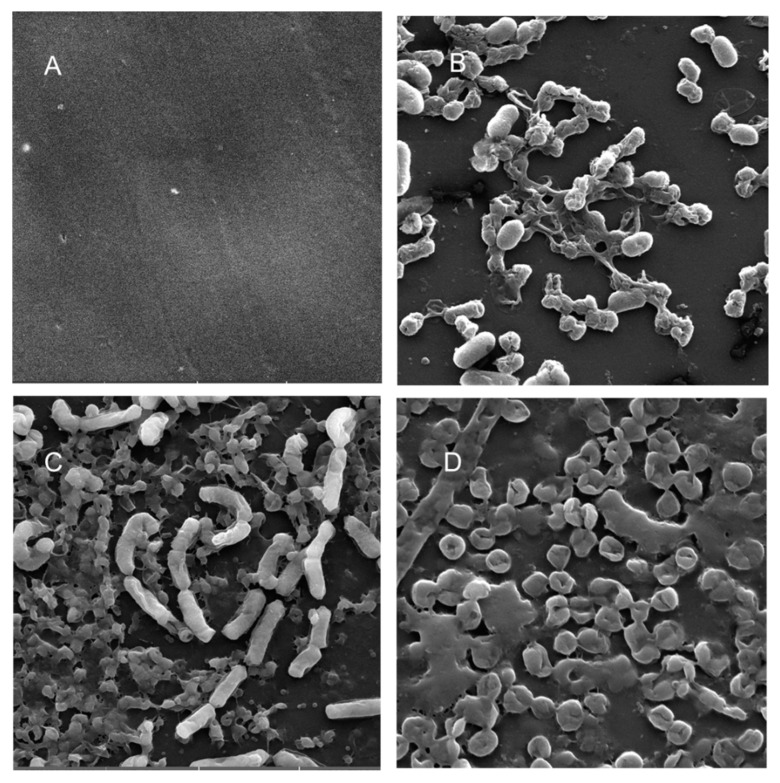
Scanning electron micrographs (**A**) of un-colonized PP (×10,000); (**B**–**D**) of *C. freundii* in the planktonic phase (×18,700), adherent phase on PP (×11,200) and biofilm phase induced on PP pressed on MacConkey agar (×18,600), respectively.

Scanning electron microscopy (SEM) was performed to compare the planktonic phases, adherent cells and blood/MacConkey media-induced classical biofilms. SEM analysis of *A. hydrophila* in the presence of PE showed that the long rod-shaped clumps of cells (Figure 5B) were transformed into long rows of cells adhered to the surface with some cell curling and the formation of early biofilm-like structures (Figure 5C). The mature biofilms induced by blood agar, on the other hand, had layers of EPS and only tiny remnants of cells visible (Figure 5D). For PP, the adherent cells had already formed EPS layers and only tiny cell fragments were visible (Figure 6C), but the blood agar induced the formation of irregularly shaped aerial structures (Figure 6D). For *C. freundii*, planktonic cells had a round morphology with clumping (Figure 7B), but the adherent cells already formed linked cells in a branching pattern and three-dimensional structures (Figure 7C). Mature biofilms induced by MacConkey medium appeared similar to the adherent cells, but were more densely networked (Figure 7D). *C. freundii* on adhered on PP differed significantly from earlier samples; here the cocci clumped together or linked by fibers (Figure 8B), are transformed into biofilms containing curled cells on top of cells trapped in EPS (Figure 8C). The MacConkey-induced mature biofilm was largely flat with cocci trapped in EPS on top (Figure 8D).

## 4. Discussion

Genome analysis of the ATCC 7966-type strain (which is closely related to RIT668) showed it to be metabolically versatile with significant virulence potential and a predicted ability to infect a variety of hosts [134]. Due to the high similarity in the genomes (99%), it is possible that *A. hydrophila* RIT668 shares this potential for broad metabolic capability, virulence and the ability to infect multiple hosts, including humans. The closest relative of RIT669 is a *C. portucalensis* strain; *C. portucalensis* strains can be multidrug resistant and some may be highly resistant livestock-origin pathogens or “superbugs” [135,136]. This raises the possibility that RIT669 may also be multidrug resistant.

Enterobactericeae, especially *Citrobacter* spp., are common among turtles, whereby immune-compromised turtles may be conducive to the expression of pathogenic potential in the gut, which should be considered during rehabilitation procedures [94]. Australian green turtles (*Chelonia mydas*) in captivity were previously shown to harbor antibiotic-resistant strains [90,94,137]. *A. hydrophila* is common to infect turtles, fish and other amphibians as well as humans, as it is widely distributed in fresh water, estuarine and marine environments [138,139]. Among turtles, *A. hydrophila* infections were reported in *Pseudemis scripta* [140] and soft-shelled turtles (*Trionyx sinensis*) [141,142,143]. In the 1994 outbreak of *A. hydrophila* in Italy, there was a 95% mortality rate and the autopsies revealed infection in visceral organs [140]. The animals in that study were apathetic, did not feed and were lethargic in their movements. Our animals were similarly sluggish and the mortality rate was 100% by the end of the study.

Previously, it was shown that healthy pet turtles from seven species (other than *Clemmys guttata*) and their environment harbored *Citrobacter* spp. that were multidrug resistant, formed biofilms and were positive for *slt-II* and *viaB* [85]. In another study, all the turtles sampled had bacteria resistant to at least two antibiotics and 24% of the isolates were resistant to seven of the eight antibiotics tested [144]. Therefore, the predicted multidrug resistance characteristics of *A. hydrophila* RIT668 and *C. freundii* RIT669 are not surprising, and this fits into the growing number of studies which suggest that both *A. hydrophila* and *C. freundii* are emerging pathogens of concern. In addition, our strains do harbor more extensive resistomes in comparison to previous studies. Bacteria associated with reptiles could cross species barriers and infect mammals, according to a study of pet green turtles in which reptile and clinical strains for some enterobacterial genera (including *Aeromonas*) were identical [83]. We suggest that zoonotic transmission risks exist for these two potential pathogens and believe that their transmission needs to be monitored from a One Health perspective.

Shiga toxins (Stx) and Shiga-like toxins (Slt) are a group of bacterial toxins involved in human and other animal diseases; they are the cause of bloody diarrhea and hemolytic uremic syndrome [145,146]. Stx or Slt toxins are produced by enterohemorrhagic *E. coli*, *Shigella dysenteriae* type 1, *C. freundii*, *Aeromonas* spp. and *Acinetobacter haemolyticus* [38,39,81,145]. Diarrhea-associated *C. freundii* isolates are reported to contain several toxins, including Shiga-like toxins, heat-stable toxins and a homolog of the cholera toxin B subunit [59]. Clinical isolates of *Aeromonas* spp. possess Shiga toxin genes (*stx*1 and *stx*2), whose sequence is highly similar to the most virulent gene variants of *E. coli* strains [38]. It is notable that other studies showed specifically that microplastics in the environment may induce the overexpression of other types of virulence-related genes like the integrase *int1* genes [98]. Similarly, the increased expression of the *slt-II* gene upon plastic colonization across all the samples suggests a possible adhesion-specific or even plastic adhesion-specific mechanism of upregulation. However, biofilm-related genes were not upregulated in this study and the lack of correlation between the expression of biofilm-related genes and the toxin genes remains unexplained. In *E. coli* O104:H4 expressing the *stx2* gene, the correlation of the expression of other genes such as *pgaA* and *aggR* with *stx2* expression is strain dependent [131]. Another study in the same *E. coli* strain, the only gene reported to be sufficient for plant surface adhesion is *ompA*, but the correlation is not strong [147]. Therefore, the co-regulated expression of biofilm and virulence-related genes is not consistent across different bacterial species, or even strains of the same species.

Electron microscopy has been used from the 1990s for the examination and characterization of biofilms on medical devices [148,149]. Scanning electron microscopy (SEM) has the level of magnification and resolution necessary to observe the overall shape of microorganisms in the biofilm, as well as their three-dimensional organization [150,151]. *Aeromonas* spp. were previously reported to attach to surfaces and eventually form biofilms, even if they were freely able to grow in water [52].

A recent study reported that floating biofilm-like structures (BLSs) and the attached biofilms had different metal resistance properties [152]. It has been suggested that “reversible” and “irreversible” attachment to a surface, as well as “surface-sentient” and “surface-naïve” planktonic cells are distinct [153]. The current work suggests that there could be different stages, and that adherent cells may have different properties from true biofilms. Thus, the adherent cells in a system with intermittent shear may not morphologically resemble the well-studied mature biofilm forms induced by blood/bile components, even though they may upregulate the expression of certain biofilm genes or toxins. Further, the structures formed in each case may differ based on both the bacterium and the polymer. Therefore, gene expression during different stages of attachment may be more nuanced than hitherto appreciated.

## 5. Conclusions

The isolated strains were shown to have significant resistomes, with *A. hydrophila* containing predicted resistance genes to six antibiotic classes and *C. freundii* containing resistance genes for 19 classes. The expression of many of the genes examined did not follow a specific pattern, since the adhesion to the polymers could be controlled via the complex interplay of several genes that were or were not included in this study. However, the clear exception is *slt-II*, whose expression is increased in response to either PE or PP for both bacteria. The toxin expression notwithstanding, electron microscopy showed that the adherent cells form structures different from well-studied biofilms growing on media with blood/bile components. Extensive antibiotic resistance repertoires, biofilm formation, colonization of common plastics and the overexpression of the *slt-II*-type diarrheal toxin in plastic-adherent cells, along with the origin in of the bacterial isolates from reptilian niches warrant the classification of both strains in this study as potential opportunistic zoonotic pathogens.

## Figures and Tables

**Table 1:** Summary of whole-genome sequencing of *A. hydrophila* and *C. freundii*.

Organism	Accession No.	Genome Size (bp)	%GC Content	Genome Coverage	No. of Contigs	No. of ORFs	No. of tRNAs	No. of rRNAs
*Aeromonas hydrophila* RIT668	JABAJN000000000	4,773,422	61.52	82X	90	4341	99	4
*Citrobacter freundii RIT669*	JABAJM000000000	4,900,040	51.97	80X	76	4645	72	4

**Table 2:** Resistance Gene Identifier (RGI) analysis for *A. hydrophila*.

Category	Read Count	16S-Normalized Read Count	RGI Criteria	Antimicrobial Resistance Gene Family	Drug Class	% Identity of Matching Region	% Length of Reference Sequence	
multidrug	72	2.65	Strict	TRU beta-lactamase	Carbapenem	96.46	100	
unclassified	4	0.35	Strict	CphA beta-lactamase	Penem, Cephalosporin	93.56	100	
beta-lactam	5	0.26	Strict	resistance-nodulation-cell division (RND) antibiotic efflux pump	Fluroquinolone, Tetracycline	49.32	98.39	
MLS (macrolide, lincosamide, streptogramin)	4	0.2	Strict	elfamycin-resistant EF-Tu	Fluroquinolone, Tetracycline	43.71	99.06	
aminoglycoside	3	0.16	Strict	elfamycin-resistant EF-Tu	Elfamycin	90.84	96.33	
bacitracin	3	0.16	Strict	OXA beta-lactamase	Elfamycin	90.84	96.33	
glycopeptide	1	0.06	Strict	resistance-nodulation-cell division (RND) antibiotic efflux pump	Cephalosporin	99.74	110.73	

**Table 3:** Resistance Gene Identifier (RGI) analysis for *C. freundii.*

Category	Read Count	16S-Normalized Read Count	RGI Criteria	Antimicrobial Resistance Gene Family	Drug Class	% Identity of Matching Region	% Length of Reference Sequence	
multidrug	72	2.6504884	Perfect	CMY beta-lactamase	Cephamycin Cephalosporin	100	100	
unclassified	4	0.351411	Strict	penicillin-binding protein mutation conferring resistance to beta-lactam antibiotics	Carbapenem, Cephamycin Penem, Monobactam, Cephalosporin	52.75	96.39	
beta-lactam	5	0.2594203	Strict	kdpDE	Aminoglycoside	90.62	100	
MLS (macrolide, lincosamide, streptogramin)	4	0.1995819	Strict	MFS, RND antibiotic efflux pump	Cephamycin; Cephalosporin; Fluoroquinolone, Macrolide, Penem	95.62	100	
aminoglycoside	3	0.1573626	Strict	ATP-binding cassette antibiotic efflux pump	Nitroimidazole	94.33	100	
bacitracin	3	0.1573626	Strict	RND antibiotic efflux pump	Macrolide, Fluoroquinolone, Penem	99.05	100	
glycopeptide	1	0.0625328	Strict	antibiotic resistance nfsA	Nitrofuran	85.8	100	
			Strict	GlpT	Fosfomycin	94.91	100	
			Strict	MFS antibiotic efflux pump	Fluoroquinolone	94.29	100	
			Strict	RND, antibiotic efflux pump	Aminoglycoside, Aminocoumarin	97.07	100	
			Strict	pmr phosphoethanolamine transferase	Peptide	87.85	101.55	
			Strict	quinolone resistance protein (qnr)	Fluoroquinolone	99.56	100	
			Strict	elfamycin-resistant EF-Tu	Elfamycin	98.75	78.24	
			Strict	major facilitator superfamily (MFS) antibiotic efflux pump	Fluoroquinolone	95.12	100	
			Strict	resistance-nodulation-cell division (RND) antibiotic efflux pump	Aminocoumarin	93.27	100	
			Strict	resistance-nodulation-cell division (RND) antibiotic efflux pump	Cephalosporin, Fluoroquinolone Phenicol Tetracycline Glycylcycline, Penem, Rifamycin Triclosan	94.57	100	
			Strict	resistance-nodulation-cell division (RND) antibiotic efflux pump	Cephalosporin, Fluoroquinolone Penem, Phenicol, Glycylcycline, Tetracycline, Rifamycin Triclosan	90.97	100	
			Strict	major facilitator superfamily (MFS) antibiotic efflux pump	Rhodamine, Tetracycline, Benzalkonium chloride	87.8	100	
			Strict	general bacterial porin with reduced permeability to beta-lactams, RND, antibiotic efflux pump, ATP-binding cassette (ABC) antibiotic efflux pump, MFS antibiotic efflux pump	Cephamycin, Cephalosporin, Fluoroquinolone Penem, Glycylcycline, Monobactam, Triclosan, Phenicol Tetracycline Carbapenem, Rifamycin	94.36	100	
			Strict	UhpT	Fosfomycin	94.82	100	
			Strict	RND, antibiotic efflux pump	Cephalosporin, Fluoroquinolone Phenicol Tetracycline Glycylcycline, Penem, Rrifamycin Triclosan	90.43	100	
			Strict	major facilitator superfamily (MFS) antibiotic efflux pump	Fosfomycin	90.89	99.51	
			Strict	general bacterial porin with reduced permeability to beta-lactams, resistance-nodulation-cell division (RND) antibiotic efflux pump	Cephamycin, Cephalosporin, Fluoroquinolone, Glycylcycline, Penem, Monobactam, Triclosan, Phenicol, Tetracycline, Carbapenem, Rifamycin	94.44	100	

**Table 4:** Gene annotations and primer sequences of the biofilm-related and virulence genes in *A. hydrophila* and *C. freundii.*

Accession Number or Locus Tag	Genes Names	Annotation	Primer Sequence (5′–3′)	T_m_ °C
KC489166	16S RNA	16S ribosomal RNA (house-keeping gene)	TTACCTACTCTTGACATC	55.0
			GACTTAACCCAACATTTC	
B2G73_RS15900	*bsmA*	biofilm peroxide resistance protein	TAATGGGTTACAGCGAATAG	53.1
			ATAAGACCACATAATAATCAGC	50.6
B2G73_RS10300	*bssR*	biofilm formation regulatory protein	CGCTTATCTGCTGTTGAG	52.9
			ATACCGTGAAGTTGTGATTG	53.5
B2G73_RS09175	*bssS*	biofilm formation regulatory protein	GGACTGAAGTTGGACAAA	51.5
			CGCTGATACTCATTTACCT	50.3
B2G73_RS19460	*hmsP*	biofilm formation regulator	GTTAATACTCACGGTAGC	45.1
			GGTAATGCCAGTTGATAG	48.5
B2G73_RS15490	*tabA*	Toxin-antitoxin biofilm protein	GTCGGCAATATTCACAAC	52.0
			TCATATCTTCGGCAATCA	53.3
B2G73_RS09280	*csgD*	transcriptional activator of curli operon	GCGTTATTACAGCACTTA	47.1
			TTATCTGCCTCCATCATAT	50.3
-	*viaB*	Virulence (Vi polysaccharide antigen)	TGTCGAGCAGATGGATGAGCAT (VIAB-1)	65.4
			ACGGCTGAAGGTTACGGACCGA (VIAB-2)	69.1
-	*slt*-ii	Virulence (SLT-II enterotoxin)	CCGGATCCATGAAGTGTATATTATTTAAATGG (GK1)	62.0
			CCCGAATTCTCAGTCATTATTAAACTGCAC (GK4)	67.2

**Table 5:** Antibiotics and Secondary Metabolites Analysis Shell (antiSMASH) prediction of biosynthetic gene clusters involved in the synthesis of antibiotics and secondary metabolites in *A. hydrophila* and *C. freundii.*

Organism	Cluster Number	Location within the Cluster	Predicted Gene Product	Percent Similarity to Known Cluster (Name of Cluster, Type)
*A. hydrophila*	6	36,891–91,228	NRPS (non-ribosomal peptide synthase)	100 (Amonabactin, NRPS)
*A. hydrophila*	27	34,062–70,902	arylpolyene	90 (*Aeromonas* sp. arylpolyene)
*A. hydrophila*	15	59,435–80,058	homoserine lactone	100 (*Aeromonas* sp. homoserine lactone)
*A. hydrophila*	37	8,045–18,305	bacteriocin	80 (*A. hydrophila* Strain TN-97-08, bacteriocin)
*C. freundii*	2	283,708–327,304	arylpolyene	94 (APE Ec biosynthetic gene cluster from *E. coli* CFT037, arylpolyene)
*C. freundii*	8	1–40,280	NRPS	30 (Turnerbactin biosynthetic gene cluster from *Teredinibacter turnerae* T7901, NRPS)
